# Effect of different types and levels of fat addition and pellet binders on physical pellet quality of broiler feeds

**DOI:** 10.3382/ps/pez190

**Published:** 2019-04-19

**Authors:** Mohammad Hossein Mohammadi Ghasem Abadi, Hossein Moravej, Mahmoud Shivazad, Mohammad Amir Karimi Torshizi, Woo Kyun Kim

**Affiliations:** 1 Department of Animal Science, College of Agriculture and Natural Resources, University of Tehran, P. O. Box 31585-4111, Karaj, Iran; 2 Department of Poultry Science, Faculty of Agriculture, Tarbiat Modares University, P. O. Box 14115-336, Tehran, Iran; 3 Department of Poultry Science, University of Georgia, Athens, GA 30602

**Keywords:** soybean oil, calcium fat powder, mixer-added fat, pellet quality, pellet binder

## Abstract

Two experiments were conducted to evaluate the effects of different types and levels of mixer-added fat (soybean oil: SO and calcium fat powder: CFP) and pellet binders (PBs: calcium lignosulfonate (CaLS) and bentonite (Ben)) on physical pellet quality (PPQ) parameters. PPQ included pellet durability index (PDI), pellet hardness, and pellet length of broiler diets processed under short-term conditioning. The first experiment had 4 treatments arranged as a 2 × 2 factorial with 2 types (SO and CFP) and 2 levels (1.5 and 3%) of mixer-added fat. In the second experiment, 22 treatments, combinations of 2 types of mixer-added fat (SO and CFP) at 3 levels (0, 1.5 and 3%) and 2 types of PB (CaLS = 0, 0.5, and 1% and Ben = 0, 1, and 2%), were arranged by a completely randomized design. PDI was measured by 2 devices: Pfost Tumbling box (PDIT) and Holmen NHP tester (PDIH). The results showed that the diets containing 1.5% CFP without PB had significant differences in all PPQ parameters. The results revealed that adding 0.5% CaLS to the 3% SO diets significantly enhanced PDIH, pellet hardness, and pellet length compared to other treatments. Moreover, 1.5% CFP diets with 2% Ben had significantly higher PDIT, PDIH, and pellet hardness among the treatments. Based on contour plots, different levels of Ben in the diets containing SO failed to create optimum PDIT values (>96%). However, 1.5 to 2.50% CFP diets without Ben had the optimum PDIT values. The optimum PDIT value was achieved by the diets containing 3% SO in the range of 0.21 to 0.56% CaLS. Furthermore, adding 0.5% CaLS to the diets containing less than 2.86% SO resulted in suboptimal PDIT values (<96%). The diets containing 1.5 to 2.50% CFP without CaLS had the optimum PDIT values. However, increasing CaLS levels more than 0.38% led to suboptimal PDIT values. Overall, these results indicated that the selection of appropriate PBs should be based on type and level of mixer-added fat.

**ABBREVIATIONS**
Ben: bentonite
CaLS: calcium lignosulfonate
PB: pellet binder
PPQ: physical pellet quality
PDI: pellet durability index
PDIT: Pfost Tumbling box PDI
PDIH: Holmen PDI
SO: soybean oil

## INTRODUCTION

Pelleting is one of the relevant hydrothermal process in poultry feed production (Abdollahi et al., 2013). Recent studies have shown that feeding pellet diets per se do not bring an optimum performance of broilers (Naderinejad et al., 2016; Mohammadi Ghasem Abadi et al., 2019). Hancock (2010) asserts that poor physical pellet quality (**PPQ**) produces more fine particles during feed transportation from feed mill to poultry house feed lines; moreover, some researchers (Quentin et al., 2004; Corzo et al., 2011; Lilly et al., 2011) report that poor PPQ negatively changed feed intake pattern of broilers. PPQ commonly measures through 3 methods: 1) pellet durability index (**PDI**) by different devices (Pfost Tumbling box [**PDIT**] and Holmen NHP series tester [**PDIH**]), 2) pellet hardness by texture analyzer devices (Khal tester or automatic texture analyzer), and 3) visual evaluation of macrostructure of pellet surface or pellet length (Winowiski, 1995; Thomas and van der Poel, 1996; Payne, 1997; Pope, 2016). It is generally believed that Pfost method leads to superior results rather than Holmen tester and has a close relationship with actual fine percentage produced by farm feed lines (Hancock, 2010; Fahrenholz, 2012). Parsons et al. (2006) study revealed that broiler fed hard pellet (18.5 Newton breaking force) had significantly better performance than soft pellet (16.2 Newton breaking force) diets during 3 to 6 wk; therefore, not only PDI but also pellet hardness is considered as an important PPQ parameter.

Feed formulation and grinding had greater impact (60%) on PPQ than other factors related to pelleting process (40%) (Behnke, 1996; Cavalcanti and Behnke, 2005; Loar and Corzo, 2011). The effects of different factors such as type and level of feed ingredient inclusion, specially dried distillers grains with solubles, dicalcium phosphate and mixer-added fat (Rigby et al., 2018), and feed milling process (feed particle size, conditioner retention time, production rate, die thickness) were investigated on PDI (Greenwood and Beyer, 2003; Fahrenholz, 2012; Muramatsu et al., 2015; Pope, 2016). Previous studies have confirmed that fine grinding resulted in better PDI (Angulo et al., 1996; Amerah et al., 2008; Chewning et al., 2012; Mohammadi Ghasem Abadi et al., 2019). Based on feed pellet quality factor introduced by Payne et al. (1994), the mixer-added vegetable oils had a negative effect (–40 value) on PPQ (Thomas et al., 2001). Stark`s (1994) study showed that there is an interaction between fat types (tallow, soybean oil [**SO**], poultry fat, and choice white grease) and levels (1.5 and 3%) on PDI. The findings of Zimonja et al. (2007) revealed that SO and saturated fat powder (65% palmitic acid: Akofeed) had different impact on PDI and implied that each type of fat had individual pellet quality factor values. However, poultry nutritionists still need fat sources for formulating the high energy density diets. The increase of fat price and detrimental effect of mixer-added fat on pellet durability have made nutritionists to reduce the amount of fat added in poultry diets (Gehring et al., 2011). Though, an increasing mixer-added fat level may reduce the total cost of broiler production due to their positive effects on conserving the heat-sensitive nutrients by coating the feed particle, die lubrication, and reduction of electrical energy of the pelleting process (Gehring et al., 2011). Post-pellet application of fat helps to increase oil inclusion in the diets. However, this method does not have enough accuracy and significantly increases the cost of diets. Furthermore, it needs highly durable and porous pellets (Fischer and Ravnsborg, 2013; Lamichhane et al., 2015). Therefore, there is a lack of research on the effect of different mixer-added fat sources (SO and calcium fat powder [CFP]) and pellet binders (**PBs**) on PPQ of conventional poultry diets processed under a short-term conditioning situation.

The feed industry has been trending towards the use of long-term conditioning (e.g., conditioner temperature = 85°C and conditioner retention time = 3 min) to improve PDI (Cutlip et al., 2008; Abdollahi et al., 2010; Attar et al., 2018) and to hygiene feed through *Salmonella* spp. population reduction (Pickford, 1992; Peisker, 2006), while it damages heat sensitive nutrients and consequently reduces nutrient digestibility (Creswell and Bedford, 2006; Kenny and Felemming, 2006; Slominiski et al,. 2007; Abdollahi et al., 2008; Krabbe et al., 2012). However, long-term conditioning is not able to eliminate the concerns of recontamination of final feed with pathogen (Peisker, 2006; Boroojeni et al., 2016). Therefore, the use of short-term conditioning (conditioner temperature = < 75°C and conditioner retention time = < 30 s) separately or in combination with organic acids to reduce the *Salmonella* spp. population (Boroojeni et al., 2014; Boney et al., 2018; Jendza et al., 2018). Loar et al., (2014) indicated that the use of long-term conditioning and a low mixer-added fat level (1%) significantly improved PDIT values. The common technical term, “good pellet,” was coined to describe the pellets with high nutrient availability and PPQ (Abdollahi et al., 2013). The confounding effect of long-term conditioning on improving PDI makes the PB inefficient to enhance PPQ (Moritz, 2014; Pope, 2016). Hence, adding PBs under short-term conditioning result in improved PPQ and lead to the production of “good pellet” (Abdollahi et al., 2012; Boney and Moritz, 2017). Different solutions have been used in order to improve PPQ. The first solution is the liquid addition technique, in which a liquid such as water can be evenly sprayed into a mixer (Moritz et al., 2003; Hott et al., 2008). Water spray into a mixer at the specific dosage is considered as the first solution for improving PPQ which is the cheapest method to enhance PDI, but it may increase the risk of mixer contamination and wet mixing time during the feed manufacturing. Moreover, it increases a risk of mold growth in pelleted feed and consequently reduces shelf life of feed (Lundblad et al., 2009). The second solution is solid PBs addition such as bentonite (**Ben**) (Salmon, 1985; Salari et al., 2006; Attar et al., 2018; Moradi et al., 2018) and calcium lignosulfonate (**CaLS**) (Acar et al., 1991; Corey et al., 2014). Therefore, the aim of this study was to evaluate the effect of different levels and types of mixer-added fat (SO and CFP) and solid PBs (Ben and CaLS) on PPQ parameters of practical corn-soybean based diets under short-term conditioning situation.

## MATERIALS AND METHODS

### Feed Formulation and Processing

In the first and second experiments, 120 kg practical corn-soybean meal finisher broiler diets for each treatment were manufactured in a feed mill located in Gorgan. The first experiment had 4 treatments consisted of 2 types (SO and CFP) and 2 levels (1.5 and 3%) of mixer-added fat with a 2 × 2 factorial arrangement. The second experiment consisted of 22 treatments, combinations of 2 types of mixer-added fat (SO and CFP) at 3 levels (0, 1.5, and 3%) and 2 types of PB such as CaLS (0, 0.5, and 1%) and Ben (0, 1, and 2%). Two sources of fat (SO and CFP) at different levels (0, 1.5, and 3%) were used for producing 5 types of diets such as the diets without fat, the diets with SO at 2 levels (1.5, and 3%) and the diets with CFP at 2 levels (1.5 and 3%) (Table 1). In the second experiment, different types of PBs (Ben: 0, 1, and 2% and CaLS: 0, 0.5, and 1%) were added to 5 diets to create 22 treatments. Poultry specific CFP (Persia fat, Qom, Iran) as a solid powder containing AMEn: 8,000 kcal/kg, calcium: 10–12%, ether extract: 85%, saturated fatty acid: 35%, unsaturated fatty acid: 65%, and glycerol: 10%. All ingredients were weighed and ground by a hammer mill (ASIAB industry factory, Tehran, Iran) with a 2.0-mm sieve screen. The batches were blended in a ribbon mixer (for dry mixing 180 s and wet mixing 90 s for SO). All PBs (activated sodium Ben: G-Bind (Paya Farayand, Khorasan Razavi, Iran) and CaLS: Borregaard (LignoTech, Umkomaas, South Africa)) were separately added on top of each batch of 120 kg and then mixed with a micromixer for 180 s. The mash feeds were processed in a single barrel (1.2 × 0.45 m = length × diameter) conditioner under short-term conditioning (10 s retention time, steam pressure of 80 PSI, discharge feed temperature of 65°C, and moisture addition of 3% to the mash diet) and pelleted by a pellet press machine (FDSP SZLH32 model, Jiangsu, China) equipped with no relief die which had the compression ratio (die effective length to diameter ratio) equal to 10 (40:4 mm). The temperature of discharged feed from a pellet press was 5°C higher than the one of conditioner feed output. The moisture of discharged feed from a counter-flow cooler was approximately 11%.

**Table 1.** Composition of finisher broiler diets with different levels and types of mixer-added fat.

	Mixer-added fat levels				
Ingredient	0%	1.5%	1.5%	3%	3%
Maize	70.36	67.36	67.36	64.37	64.37
Soybean meal 44% CP	26.05	27.49	27.49	28.93	28.93
Soybean oil	0	1.5	0	3	0
Calcium fat powder (CFP)^1^	0	0	1.5	0	3
Dicalcium phosphate	1.38	1.42	1.42	1.46	1.46
Limestone	0.94	0.95	0.95	0.97	0.97
Sodium hydrochloride	0.25	0.25	0.25	0.25	0.25
Sodium bicarbonate	0.12	0.13	0.13	0.14	0.14
Vitamin premix^2^	0.25	0.25	0.25	0.25	0.25
Mineral premix^3^	0.25	0.25	0.25	0.25	0.25
L-Lys HCL	0.16	0.14	0.14	0.13	0.13
DL-Met	0.21	0.22	0.22	0.23	0.23
L-Thr	0.03	0.03	0.03	0.02	0.02
Calculated chemical analysis^4^					
AMEn poultry (kcal/kg as is)	2,955	3,018	3,006	3,083	3,059
Crude protein (%)	17.60	17.98	17.98	18.36	18.36
Calcium (%)	0.77	0.79	0.93	0.81	1.08
Available phosphor (%)	0.34	0.35	0.35	0.35	0.35
Digestible Lys (%)	0.91	0.92	0.92	0.94	0.94
Digestible Met+Cyc (%)	072	0.73	0.73	0.75	0.75
Digestible Thr (%)	0.59	0.60	0.60	0.62	0.62

^1^Poultry specific calcium fat powder (AMEn: 8,000 kcal/kg, calcium: 10–12%, ether extract: 85%, Saturated fatty acid: 35%, unsaturated fatty acid: 65%); Persiafat, Iran.

^2^Supplied per kg diet: vitamin A: 9,000 IU; vitamin D3: 2,000 IU; vitamin E: 18 IU; vitamin K3: 2 mg; vitamin B1: 1.8 mg; vitamin B2: 6.6 mg; vitamin B3: 10 mg; vitamin B5: 30 mg; vitamin B6: 3 mg; vitamin B9: 1 mg; vitamin B8: 0.1 mg; vitamin B12: 0.015 mg; choline: 250 mg; and antioxidant: 100 mg.

^3^Supplied per kg diet: Mn: 99.2 mg; Fe: 50 mg; Zn: 84.7 mg; Cu: 10 mg; I: 1 mg; Se: 0.2 mg; and choline: 250 mg.

^4^All values are based on NIR analytical report of Evonik Nutrition & Care GmbH.

### Physical Pellet Quality Analysis

Five kilograms from middle parts of each batch of pelleted feeds was collected and sieved by 3.150 mm analytical sieves (Eckhardt DIN 4188, Hann, Germany) and kept on nylon bags for further physical quality tests. Four replicates recorded for all PPQ parameters except for pellet hardness with 12 replicates. PDIT values were measured by a Pfost Tumbling box (ASAE, 1997). In brief, a test sample of 500 g intact pellet (without dust) was placed in a tumbling box. After tumbling for 10 min at 50 rpm in a dust-tight enclosure box, the samples were removed and sieved. The PDIT percentages were calculated by dividing the weight of intact pellet after tumbling by the weight of initial samples before tumbling (500 g) multiplied by 100. Holmen NHP 100 portable tester (Takpro Ltd, UK) was used for measuring PDIH. Briefly, a test sample of 100 g intact pellet (without dust) was placed in Holmen tester. After working for 60 s at 68 mbar pressure, the sample was removed and sieved. The PDIT percentage was calculated by dividing the weight of intact pellet after tumbling by the weight of initial sample before tumbling (100 g) multiplied by 100. Pellet hardness (as Newton) was measured by Brookfield CT3 10,000 g texture analyzer (Middleborough) with a cylindrical probe number 3. Pellets with the same length size (1 cm) were selected, and then the texture analyzer machine was programed to compress the pellet diameter up to 2 mm. The speed of probe was adjusted at 1.5 mm/s. Pellet length was measured by the described method of Winowiski (1995). Briefly, 10,000 mg of intact pellet was weighted, the numbers of full diameter (4 mm) pellet pieces were counted, and then the average weight per piece was calculated and expressed as mg per pellet.

### Statistical Analysis

All data were analyzed by Proc GLM procedure of SAS software (2004, SAS 9.1, Cary, NC). Four treatments were analyzed by the completely randomized design with 2 × 2 factorial arrangement consisted of 2 fat sources and 2 fat levels. A total of 22 treatments were analyzed by a completely randomized design. Differences among treatments were investigated by Tukey`s multiple comparison test at *P* < 0.05. The regression equation and Pearson correlation between PDIT and PDIH and contour plot figures were designed by Minitab18 statistical software (released for windows, State College, PA).

## RESULTS

The impact of different types and levels of mixer-added fat is shown in Table 2. Significant interactions (*P <* 0.05) between sources and levels of fat for all PPQ parameters were observed. Inspection of interactions revealed that the diets containing 1.5% CFP had the highest PPQ values compared to other treatments. The diets containing 3% CFP had higher (*P* < 0.05) PDI than the diets containing 3% SO (95.6 vs. 94.4% PDIT and 69.1 vs. 56.9% PDIH). The effect of different types and levels of mixer-added fat and PBs is shown in Table 3. Compared to control group (without Ben), increasing Ben level improved (*P <* 0.05) PDIH and pellet hardness values of the diets containing 1.5% SO and improved (*P <* 0.05) only pellet hardness of the diets containing 1.5% CFP. Increasing Ben levels from 1 to 2% in the diets with 3% SO and CFP cannot prevent PDI reduction. The diets containing 1.5% CFP with or without Ben had no significant PDI difference with each other. Significant PDIT differences (*P <* 0.05) were observed between 1.5% CFP diets with 2% Ben and no mixer-added fat diets with 2% Ben.

**Table 2.** The effect of 2 sources and levels of mixer-added fat on PPQ parameters.

Item		PPQ parameters			
Fat source	Fat level (%)	PDIT^1^ (%)	PDIH^2^ (%)	Pellet hardness^3^ (N)	Pellet length^4^ (mg/pellet)
SO	1.5	94.5^c^	68.9^b^	31.5^b^	111^b^
SO	3	94.4^c^	56.9^c^	24.1^c^	93^c^
CFP	1.5	96.6^a^	84.6^a^	43.2^a^	121^a^
CFP	3	95.6^b^	69.1^b^	26.1^c^	94^c^
SEM^5^		0.13	1.15	0.94	0.60
Main effects					
Fat source					
SO		94.5	62.9	27.8	102
CFP		96.1	76.8	34.6	107
SEM		0.27	2.36	0.67	1.25
Fat level					
	1.5	95.6	76.7	37.3	116
	3	95.0	63.0	25.1	93
	SEM	0.27	2.36	0.67	1.25
Probabilities, P≤					
Fat source		0.001	0.001	0.001	0.001
Fat level		0.003	0.001	0.001	0.001
Fat source × fat level		0.008	0.05	0.001	0.001
Coefficient of variation (CV%)		0.24	2.85	5.24	1.00

^a–c^Different superscript letters indicate significant differences between treatments (*P* < 0.05).

^1^Pellet durability index (PDI) based on Pfost tumbling box, 4 replicates.

^2^Pellet durability index (PDI) based on Holmen NHP100, 4 replicates.

^3^Pellet hardness based on Brookfiled CT3, 12 replicates.

^4^Pellet length based on procedure of Winowiski (1995), 4 replicates.

^5^Standard error mean.

**Table 3.** The effect of types and levels of mixer-added fat and PBs on PPQ parameters.

Treatment arrangement				PDIT^1^ (%)	PDIH^2^ (%)	Pellet hardness^3^ (N)	Pellet length^4^ (mg/pellet)
Fat type	Fat level (%)	PB type (%)	PB level (%)				
Without	0	0	0	94.73^f,g^	59.16^h^	32.52^f,g^	110.96^f,g^
Without	0	Ben	2	96.00^b,c,d^	82.86^a,b,c^	40.63^b,c,d^	123.23^c,d^
SO	1.5	0	0	94.53^f,g^	68.96^g^	31.51^g^	111.86^f,g^
SO	3	0	0	94.46^g^	56.90^h^	24.12^h^	93.06^i^
SO	1.5	Ben	1	94.66^f,g^	74.10^e^^,f^	40.24^c,d^	114.36^e^^,f^
SO	1.5	Ben	2	95.26^d,e,f^	80.43^b,c,d^	45.15^b^	113.73^e,f^
SO	3	Ben	1	95.33^d,e,f^	65.73^g^	33.29^f,g^	106.00^h^
SO	3	Ben	2	89.73^j^	22.20^k^	24.32^h^	87.03^j^
SO	1.5	CaLS	0.5	93.33^h^	76.73^d,e^	17.74^i^	109.03^g,h^
SO	1.5	CaLS	1	94.93^e,f,g^	78.73^c,d,e^	9.54^i^	126.93^c^
SO	3	CaLS	0.5	96.26^a,b,c^	85.73^a^	53.50^a^	144.63^a^
SO	3	CaLS	1	91.73^i^	38.40^i^	31.00^g^	96.43^i^
CFP	1.5	0	0	96.66^a,b^	84.60^a,b^	43.24^b,c^	121.36^d^
CFP	3	0	0	95.66^c,d,e^	69.13^f,g^	26.11^h^	94.00^i^
CFP	1.5	Ben	1	96.26^a,b,c^	84.53^a,b^	53.49^a^	133.12^b^
CFP	1.5	Ben	2	96.86^a^	86.13^a^	55.80^a^	126.03^c^
CFP	3	Ben	1	90.06^j^	19.20^k^	36.24^d,e,f^	78.53^k^
CFP	3	Ben	2	91.60^i^	29.70^j^	25.20^h^	96.33^i^
CFP	1.5	CaLS	0.5	95.20^d,e,f,g^	75.06^e^	35.38^e,f,g^	116.10^e^
CFP	1.5	CaLS	1	88.40^k^	66.82^g^	25.03^h^	110.87^f,g^
CFP	3	CaLS	0.5	95.00^e,f,g^	59.16^h^	32.34^f,g^	96.26^i^
CFP	3	CaLS	1	94.60^f,g^	65.60^g^	39.17^c,d,e^	109.03^g,h^
SEM^5^				0.13	0.80	0.75	0.60
*P*-value				<0.0001	<0.0001	<0.0001	<0.0001
Coefficient of variation (CV %)				0.29	2.46	4.37	1.10

^a–k^Different superscript letters indicate significant differences between retirements (*P* < 0.05).

^1^Pellet durability index (PDI) based on Pfost tumbling box, 4 replicates.

^2^Pellet durability index (PDI) based on Holmen NHP100, 4 replicates.

^3^Pellet hardness based on Brookfiled CT3, 12 replicates.

^4^Pellet length based on procedure of Winowiski (1995), 4 replicates.

^5^Standard error mean.

In general, the significant correlation (*P <* 0.05) was found between PDIT and PDIH data (r = 76.3%) and the linear regression equation was: PDIT (%) = 88.413 + 0.088 PDIH (%) (R^2^ = 58.2%, *P <* 0.05). Moreover, the diets containing 1.5% CFP (1 and 2% Ben) and 3% SO (0.5% CaLS) had the hardest pellet texture among the rest of treatments (*P <* 0.05). Furthermore, pellet length values of the diets containing 3% SO (0.5% CaLS) had the heaviest pellet weight (144 mg/pellet) compared to other treatments. Therefore, based on 3 PPQ parameters (PDIT, PDIH, and pellet hardness), the best treatment was the diet containing 1.5% CFP (2% Ben). However, according to PDIH, pellet hardness, and pellet length, the top treatment was the diet containing 3% SO (0.5% CaLS). For pellet hardness and pellet length parameters, there was not a significant difference between the diets containing 1.5% CFP (0 and 2% Ben).

**Figure 1.**
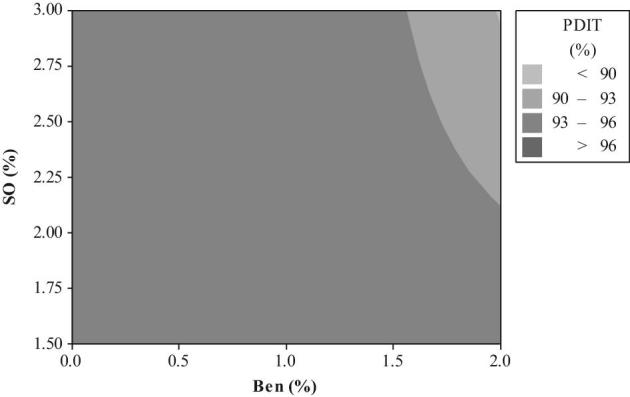
The contour plot of different levels of Ben and SO on PDIT %.

**Figure 2.**
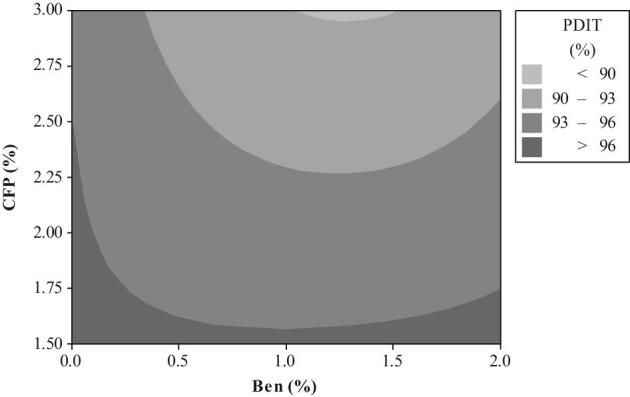
The contour plot of different levels of Ben and CFP on PDIT %.

**Figure 3.**
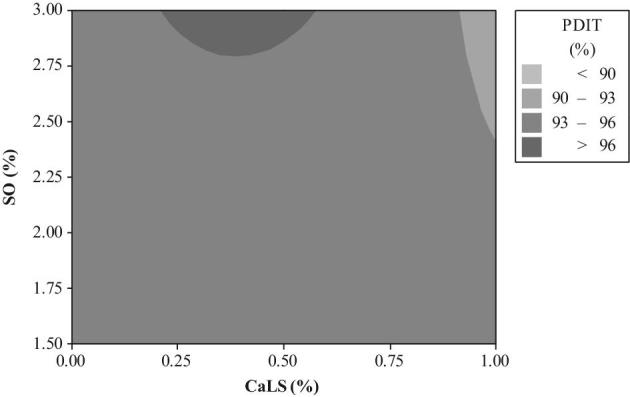
The contour plot of different levels of CaLS and SO on PDIT %.

**Figure 4.**
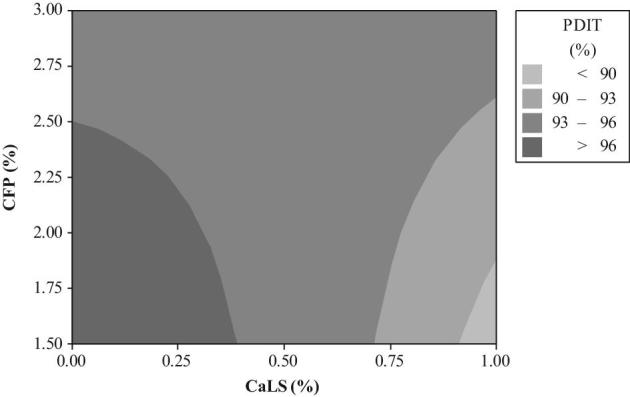
The contour plot of different levels of CaLS and CFP on PDIT %.

The relationship between a response variable (PDIT) and 2 predictor variables (2 types of mixer-added fat and PBs) was analyzed using a graphical technique of contour plot (Figures 1 to 4). The different levels of Ben in diets containing 1.5 to 3.0% SO were unable to create PDIT values up to 96% (Figure 1). However, these PDIT values were achieved by the diets with 1.5 to 2.50% CFP without any Ben (Figure 2). Regarding to an interaction between different levels of mixer-added fat and CaLS, the highest PDIT value (>96%) was obtained by the diets containing 3% SO with the range of 0.21 up to 0.56 CaLS (Figure 3). Moreover, adding 0.5% CaLS to the diets containing less than 2.86% SO reduced the PDIT value to lower than 96%. PDIT values up to 96% were achieved by the diets with 1.5 to 2.50% CFP without any CaLS (Figure 4). However, in the diets with the same levels of CFP, adding up to 0.38% CaLS reduced the PDIT (93 to 96%).

## DISCUSSION

As expected, the types and levels of mixer-added fat had a different significant effect on PPQ parameters. As the levels of SO increased in the diets without PB, all PPQ parameters except the PDIT values were significantly decreased (*P <* 0.05). Adding 1.5% CFP increased PDI in comparison to the control diet (without mixer-added fat and PB). PDIT reduction was observed in the diets containing 3% CFP without PB in comparison to the diets containing 1.5% CFP without PB. Increasing SO and CFP levels from 1.5 to 3% in diets without PB decreased PDIH by 12 percentage points and 15 percentage points, respectively. Our results were consistent with Pope (2016), who indicated that increasing SO levels from 1.5 to 3% reduced PDIH to 21 percentage points. In addition, Loar et al. (2014) observed a significant reduction of PDIT (13 percentage points) as the mixer-added fat level increased from 1 to 2.18%. Although Cavalcanti and Behnke (2005) indicated that increasing SO reduced starch gelatinization, Muramatsu et al. (2014) reported that starch gelatinization increased quadratically up to 3.5% SO. However, the results of previous studies (Moritz et al., 2003; Svihus et al., 2004; Zimonja, 2009) imply that starch gelatinization is not the major contributor to PPQ. Cavalcanti and Behnke (2005) denote that higher oil inclusion reduces frictional heat through reduction of compression forces and consequent flow of plasticized material over each other within the die orifice.

Some authors (Kersten et al., 2005; Kaliyan and Morey, 2009) reported that there are 5 binding mechanisms or adhesive forces for PPQ: 1) attraction forces between solid particles (molecular, electrostatic, and magnetic forces), 2) interlocking bound (produce from fiber, flat-shape, and bulky particles), 3) solid bridges (due to crystallization of some ingredients, chemical reaction, hardening of PBs, and solidification of melted components which mainly happens after cooling process), 4) interfacial and capillary pressure (mutual attraction of particles from surface tension of liquid bridges, due to the presence of free moisture between particles), and 5) adhesion and cohesion forces (produce from addition of highly viscous PBs such as sugar beet molasses). Our finding showed that CFP had a positive effect on PDIT in comparison to SO in diets without PB. These results were congruent with Zimonja et al. (2007), who evaluated 2 types of mixer-added fat (SO and saturated fat powder contains 65% palmitic acid: Akofeed) and 2 levels (2.5 or 5% mixer-added fat) on PDI of broiler diet. These authors explained that the inclusion of Akofeed fat increased starch gelatinization and improved PDI results against SO. It has been proved that SO creates layer-barrier problems and consequently reduces starch granules gelatinization, while a relatively higher melting point of CFP than SO allows penetration of steam into feed component before it melts (Zimonja et al., 2007). In fact, the creation of solid bridges as one of the binding mechanisms probably enhanced the PPQ of the diets containing CFP (Kaliyan and Morey, 2009). In line with previous research (Groesbeck et al., 2008; Tavernari et al., 2013), the presence of glycerol in CFP (approximately 10%) which acts as emulsifier or surfactant can improve PDIT. Recently, Cheah et al. (2017) revealed that the addition of synthetic glycerol (0.05% in the diet) as an emulsifier significantly enhances moisture content of discharged feed at a conditioner (*P <* 0.0011), feed starch gelatinization (*P <* 0.0001), and PDIT. Therefore, it seems that CFP possesses emulsifier properties compared to SO-based diets which reduce layer-barrier problem and probably leads to better starch gelatinization.

Fragmentation and abrasion are 2 phenomena that reduced PPQ (Thomas and van der Poel, 1996). PDI is one of PPQ parameters used for evaluation of abrasion and fracture of pellet into smaller particles and fines at the fracture area (Muramatsu et al., 2015). The CV and PDI difference between PDIT and PDIH (Tables 2 and 3) confirmed that Holmen tester creates a harsher challenge than Pfost method (Winowiski, 1998). Pope (2016) indicated that Pfost and Holmen testers simulate and mimic feed delivery system in the United States and Europe, respectively. Indeed, these methods were specifically designed based on different feed transportation situation from feed mills to farms in Europe and the United States (Pope, 2016). However, it is generally believed that the Pfost method leads to superior results than the Holmen tester and also has a close relationship with actual fine percentage produced by farm feed lines (Hancock, 2010; Fahrenholz, 2012). Moreover, some researchers (Salas-Bringas et al. 2007; Singh et al., 2014) reported Holmen NHP100 tester as an unreliable device. Salas-Bringas et al. (2007) mentioned that the most portion of dust remains inside the apparatus since it has one strainer and unable to describe the PPQ after a long transportation. Furthermore, pellets should have sufficient pellet hardness to avoid pellet break down due to pressure in bulk bins during storage (Major, 1984). Thus, precise PPQ evaluation needs both parameters (PDIT and pellet hardness).

Our results indicate that 2% Ben in the diets with high fat level is not sufficient to prevent PDI reduction. In fact, Ben has a water holding capacity and volume increasing properties (Moran, 1989) which increases PDI (Moradi et al., 2018). Attar et al. (2018) suggested that a higher dosage of Ben (>1.5%) may be required in a commercial situation in high energy density diets such as broiler finisher diets.

The diets containing 3% SO and 0.5% CaLS had the highest PDIH, pellet hardness, and pellet length values. Our results showed that addition of 0.5% CaLS improved PDIH by 28 percentage points in 3% SO diets and only 7 percentage points in 1.5% SO diets in comparison to the diets without CaLS. This finding was compatible with Pope (2016), who reported the greater impact of 0.5% CaLS in diets containing 3% SO than 1.5% SO (22 percentage points improvement in PDIH vs. 11 percentage points). The reason of ineffectiveness of CaLS on enhancing PPQ of diets with different levels of CPF in comparison to control diets (0% PB) is unclear. However, it seems that repulsive forces between calcium ions of solid particles may interfere with each other or water absorption competition exists between these substances when the feed passes through the conditioner.

Furthermore, Corey et al. (2014) reported that inclusion of 0.5 and 1% CaLS to the diets containing 1% SO had no significant effect on PDI compared to control group. However, addition of 0.5% CaLS to the diets containing 3% SO improved PPQ (Corey et al., 2014; Wamsley and Moritz, 2014; Pope, 2016). Pope (2016) indicated that the impact of 0.5% CaLS was magnificent under marginal pelleting condition like short-term conditioning. CaLS is a water-soluble powder, which is able to penetrate into the hydrophobic layer of feed particles and induce enough hydrogen bonding at the outer surface of the pellet (Pope, 2016). The contour plot (Figure 3) showed that the optimum BP properties of CaLS were observed from 2.86% SO, whereas Winowiski (2015) revealed that addition of 0.5% CaLS can prevent negative impact of 2% mixer-added fat on PPQ. The different responses of each PB to various ranges of SO and CFP disclosed that PBs should be chosen in accordance with types and levels of mixer-added fat.

## CONCLUSION

This study showed a positive role of adding 1.5% CFP in diets without PB in comparison to different levels of SO on all PPQ parameters. The diets containing 3% SO and 0.5% CaLS had the best PPQ values (based on PDIH, pellet hardness, and pellet length parameters). Based on PDIT, PDIH, and pellet hardness parameters, the best treatment was 1.5% CFP with 2% Ben. On the other hand, in respect to economical assessment of broiler diets and practical fat dosage used in feed manufactures (1.5% mixer-added fat), it is possible to achieve an optimum PDI value (based on PDIT and PDIH) by using only 1.5% CFP. In conclusion, the current study suggested that selection of PB should be based on type and level of mixer-added fat sources, particularly in short-term conditioning condition.
